# Retraction: Measuring the Effect of Smoking or Tobacco Use on Vertigo Among the Adult Population in the Kingdom of Saudi Arabia

**DOI:** 10.7759/cureus.r73

**Published:** 2023-06-26

**Authors:** Mohahmmed Alateeq, Tamara A Hafiz, Osama Alnizari

**Affiliations:** 1 Otolaryngology-Head and Neck Surgery, University of Hail College of Medicine, Hail, SAU; 2 Public Health, Umm Al-Qura University, Makkah, SAU; 3 Family Medicine, University of Hail College of Medicine, Hail, SAU

This article has been retracted by the Cureus editorial department due to major concerns with the methodology (including but not limited to concerns with the inclusion and exclusion criteria, use of SPSS, confusion regarding qualitative vs quantitative variables) that, in the view of the journal, affects the results and conclusions and calls into question the accuracy of the study. The authors do not agree with the decision to retract, but given the aforementioned concerns that call into question the study's accuracy, the journal has made the decision to retract without author consent. The journal greatly regrets that these issues were not identified during peer or editorial review prior to publication.

